# Design and system evaluation of a dual-panel portable PET (DP-PET)

**DOI:** 10.1186/s40658-021-00392-5

**Published:** 2021-06-12

**Authors:** Tianyi Zeng, Jiaxu Zheng, Xinyuan Xia, Xin Chen, Beien Wang, Shuangyue Zhang, Adam Chandler, Tuoyu Cao, Lingzhi Hu, Qun Chen, Xu Chu

**Affiliations:** 1 Shanghai Advanced Research Institute, Chinese Academy of Sciences, Shanghai, 201210 China; 2 University of Chinese Academy of Sciences, Beijing, 100049 China; 3 Shanghai United Imaging Healthcare Co., Ltd., Shanghai, 201807 China; 4 United Imaging Healthcare, America, Houston, TX 77054 USA

**Keywords:** PET insert, Potable PET, MR-compatible PET, System performance

## Abstract

**Background:**

Integrated whole-body PET/MR technology continues to mature and is now extensively used in clinical settings. However, due to the special design architecture, integrated whole-body PET/MR comes with a few inherent limitations. Firstly, whole-body PET/MR lacks sensitivity and resolution for focused organs. Secondly, broader clinical access of integrated PET/MR has been significantly restricted due to its prohibitively high cost. The MR-compatible PET insert is an independent and removable PET scanner which can be placed within an MRI bore. However, the mobility and configurability of all existing MR-compatible PET insert prototypes remain limited.

**Methods:**

An MR-compatible portable PET insert prototype, dual-panel portable PET (DP-PET), has been developed for simultaneous PET/MR imaging. Using SiPM, digital readout electronics, novel carbon fiber shielding, phase-change cooling, and MRI compatible battery power, DP-PET was designed to achieve high-sensitivity and high-resolution with compatibility with a clinical 3-T MRI scanner. A GPU-based reconstruction method with resolution modeling (RM) has been developed for the DP-PET reconstruction. We evaluated the system performance on PET resolution, sensitivity, image quality, and the PET/MR interference.

**Results:**

The initial results reveal that the DP-PET prototype worked as expected in the MRI bore and caused minimal compromise to the MRI image quality. The PET performance was measured to show a spatial resolution ≤ 2.5 mm (parallel to the detector panels), maximum sensitivity = 3.6% at the center of FOV, and energy resolution = 12.43%. MR pulsing introduces less than 2% variation to the PET performance measurement results.

**Conclusions:**

We developed a MR-compatible PET insert prototype and performed several studies to begin to characterize the performance of the proposed DP-PET. The results showed that the proposed DP-PET performed well in the MRI bore and would cause little influence on the MRI images. The Derenzo phantom test showed that the proposed reconstruction method could obtain high-quality images using DP-PET.

## Background

Positron emission tomography (PET) is a biomedical imaging modality which can provide 3D molecular and functional images by detecting high-energy photons generated by the positron and electron annihilation [1–3]. Thanks to the high sensitivity and exquisite specificity of targeted radioactive tracer, PET has been widely used in clinical settings for the diagnosis of cancer, neurodegenerative disease, and cardiovascular disease [4]. PET is usually performed in combination with other imaging modalities which can offer anatomical structures for localization. Currently, there are two types of commercially available hybrid PET systems including PET/CT (X-ray computed tomography) and integrated PET/MR (magnetic resonance). Although integrated PET/MR is being clinically adopted at a slower pace compared to PET/CT, it is demonstrated as a powerful multimodality imaging tool in medical research and in clinical practice [5]. Compared with CT, MRI features unique advantages in revealing anatomical morphology and physiological function information with superior soft tissue contrast. Furthermore, since there is no ionizing radiation in MRI, it leads to lower radiation doses for pediatric patients and other radiation-sensitive patient groups. These features make PET/MR a preferred choice compared with PET/CT in certain clinical applications such as brain imaging, cardiac imaging, and breast imaging.

The integration of PET and MRI is technically challenging, mainly because of the mutual interference between the two subsystems [6]. The major hardware challenges are the electromagnetic compatibility of PET detectors in a strong magnetic field environment, radio frequency interference with magnetic resonance, gradient pulse-induced eddy current, and additional field inhomogeneity of the main magnetic field introduced by ferromagnetic PET components [7–10]. The image reconstruction for PET/MR also faces unique challenges compared to PET/CT, including MR-based attenuation correction [11], truncation compensation [12], and MR-based motion correction [13].

The continuing effort to integrate PET and MRI began in the 1990s. In 1997, Shao et al. placed scintillation crystals in a 0.2-T MR imaging system [14] and connected them to the photomultiplier tubes (PMTs), the light sensors, located outside of the MR magnetic field by means of a long optical fiber to conduct PET/MR imaging research on phantoms. With the development of MR-compatible PET detector, integrated whole-body PET/MR technology continues to mature and is now broadly adopted in clinical settings [15–18]. However, due to the special design architecture, integrated whole-body PET/MR comes with a few inherent limitations. Firstly, whole-body PET/MR lacks sensitivity and resolution for focused organs, e.g., brain and breast, which limits its further clinical application such as image-guided biopsy and T-staging. Secondly, broader clinical access of integrated PET/MR has been significantly restricted due to its prohibitively high cost [19]. The MR-compatible PET insert is an independent and removable PET scanner, which has been designed to function properly in the presence of a strong magnetic field and requires minimal hardware modification to the MRI system. There have been a few initiatives to develop MR-compatible PET inserts [20–23], which show great potential for their future clinical adoption. A few successful prototypes have been developed for dedicated brain and small animal imaging applications using radiofrequency (RF)-shielding techniques and RF-penetrable technology [19, 24, 25]. However, all existing MR-compatible PET insert prototypes require cumbersome power supply systems and cooling systems, and hence, mobility and configurability of these systems remain limited.

With the intention to further enhance flexibility and sensitivity while reducing system complexity, we developed a novel portable MR-compatible PET insert, which features novel carbon fiber shielding, battery power supplies, and a phase-change cooling system. We verified its performance in a dual-panel configuration—the DP-PET (dual-panel portable PET), ideal for breast imaging. A GPU-based reconstruction method with resolution modeling was developed for the DP-PET reconstruction. In this paper, we present the system design, reconstruction, and characterization of the prototype MR-compatible DP-PET. PET performance as well as the mutual interaction between PET and MRI was also investigated.

## Methods

### System design and specification

The MRI compatible and portable PET insert, DP-PET, consists of two panel detector modules each encased in carbon fiber Faraday cage. We define the system coordinate as follows: x, vertical direction of DP-PET; y, MRI axial direction; and z, the direction perpendicular to the two parallel detector panels.

Each detector module contains 5 × 7 blocks with 7 blocks along the y direction, while each block has 4 SiPM detector readouts coupled to an array of 7 × 8 15.5 × 2.76 × 2.76 mm^3^ LYSO crystals. The internal light guide of the crystals is a proprietary design, and the modules were manufactured by United Imaging Healthcare Co., Ltd. (UIH), Shanghai. The entire DP-PET system has 3920 crystals, with each panel containing 35 crystals in the x direction and 56 crystals in the y direction. The total dimension of each panel is 370 mm (y) by 110 mm (x). The panel spacing is variable, but for this study, we set it to 160 mm as this is the typical width of a pendant breast. The dimension of the FOV is 100 × 160 × 160 mm^3^, with the central FOV offset defined as (0, 0, 0) mm.

**Fig. 1:**
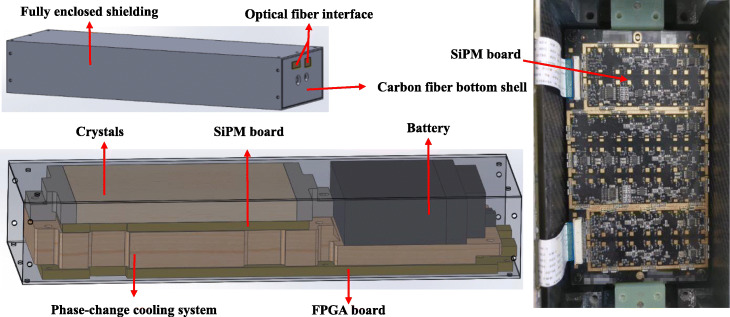
Mechanical structure of one detector module (left) and real picture of SiPM board (right)

Figure 1 shows the mechanical structure for one of the detector modules from the DP-PET insert. Data from the SiPMs are read out and pre-processed by a field programmable gate array (FPGA) on the FPGA board, which also contains an optical transceiver synchronized with the opposite detector module (through an optical fiber). There are 3 ADCs on each FPGA board with 8 channels at 80 Msps. The pre-processed data is formatted and transmitted via a high-speed optical fiber. Two regular fiber transceivers are connected to an acquisition board that is mounted on a PC via a PCIe interface at a 1.5-Gbps data rate. The *Single event* data is then converted to *Coincidence event* data through an offline coincidence sorting module, with a 4-ns timing window and a 430–600-keV energy window. The FPGA board is powered by a set of 2 MR-compatible battery packs in series (16,000 mAh), and the 30-V bias voltage of the SiPM board is powered by a set of 8-battery packs in series (16,000 mAh). With the power of the whole system being 10 W, DP-PET can operate for 60 min with fully charged battery packs.

**Fig. 2:**
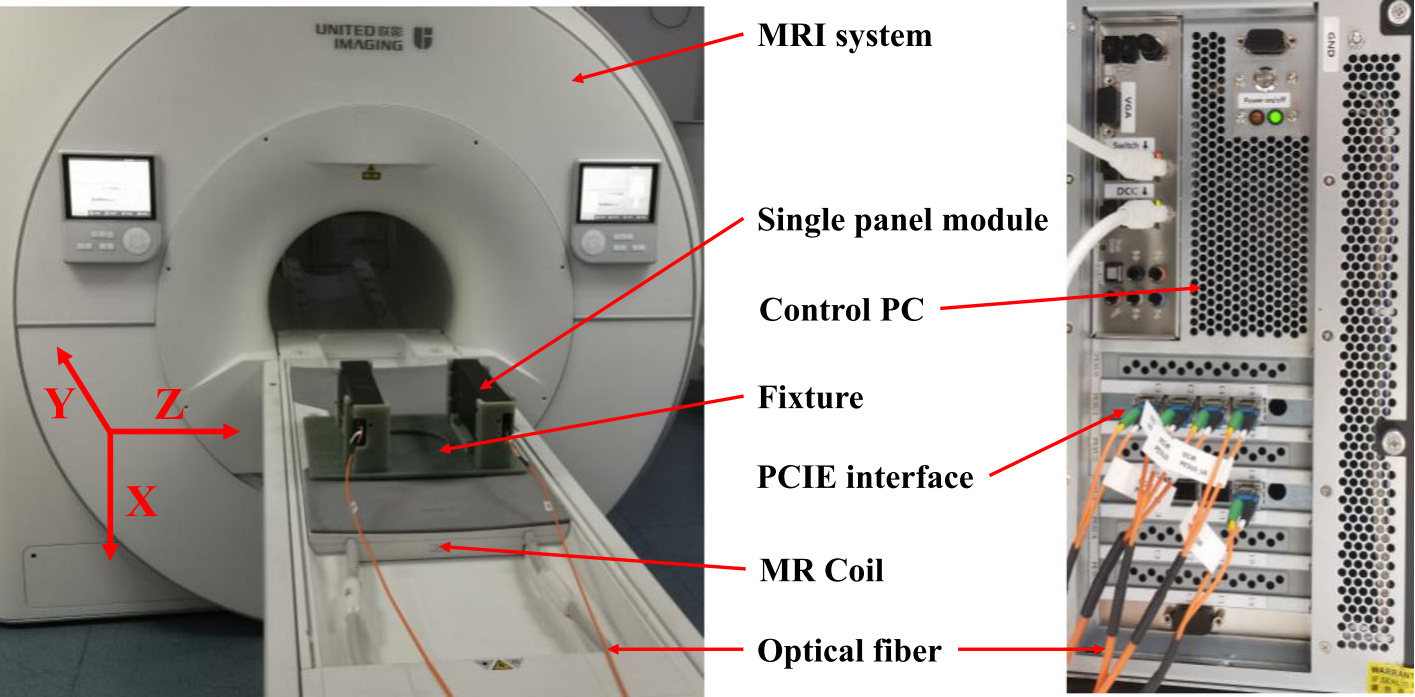
Picture of DP-PET-inserted MR system

The mechanical support and shielding structure of the DP-PET are designed to effectively shield electromagnetic waves from the MRI system. In order to eliminate the eddy current generated by the shielding shell, we used carbon fiber shielding to replace the traditional metal shielding. The surface of the carbon fiber material is processed via explosive spraying technology to form a 20-μm-thick coating. There are lines and irregular patterns on the coating, which further reduce the low-frequency conductivity of the shell to eliminate eddy currents. In addition, soft conductive materials were contained at the joints to increase sealing performance. Since there are no large areas or large metal in the whole DP-PET scanner, it presents good electromagnetic compatibility with the MRI system. Figure 2 shows the prototype DP-PET system mounted on the patient bed of a clinical 3-T MRI system (uMR790, UIH, Shanghai).

**Fig. 3:**
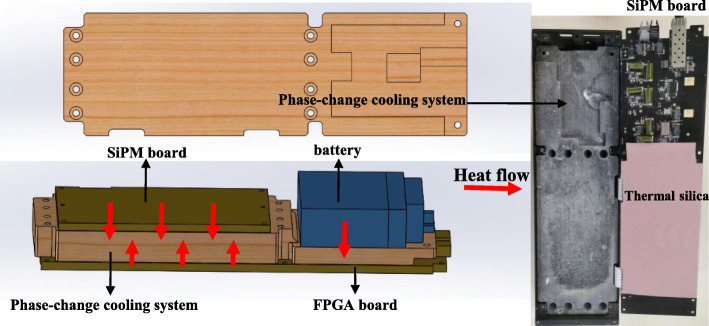
Schematic of the phase-change cooling system and picture of the FPGA board with cooling system

Considering the flexibility and portability, the water-cooling system which is commonly used in commercial PET scanners was replaced by a phase-change cooling system in the DP-PET. Figure 3 shows the schematic of the phase-change cooling system. The phase-change cooling system is composed of phase-change cooling material and thermal silica. This material absorbs a large amount of heat when the rising temperature caused by phase-changes occurs so it can maintain the temperature near the phase-change point [26, 27]. The cooling system was attached to the SiPM board, FPGA board, and batteries which are prone to generate heat during operation. After connecting with the cold source, it can quickly release heat through thermal silica and be reused.

Before reconstruction, the list-mode data was firstly transferred to panelgram (sinogram of dual-panel systems) data. The crystal IDs of the coincidence crystal pair were read from list-mode data and were counted in the panelgram. The output panelgram had a dimension of 35 × 56 × 35 × 56, with a total of 3,841,600 elements, which occupied approximately 29 MB of computer memory. A GPU-accelerated iterative image reconstruction method was developed based on the maximum likelihood expectation maximization (MLEM) algorithm and point spread function (PSF) resolution modeling [28]. The GPU kernel function was implemented to compute the forward projection and back projection in the line-of-response (LOR) units. The normalization correction matrix was acquired by scanning a plane source of 155 × 90 × 5 mm^3^ filled with ^18^F-FDG and placed parallel to the two detector panels. The normalization correction matrix was first back projected to precompute a sensitivity map. Some modifications were applied to the reconstruction method in order to better fit the DP-PET. A tube-of-response (TOR)-based ray-tracing algorithm, in which the sum of the cross-sections between the voxels, dubbed TOR, instead of the line integrals of LORs was calculated [29] replacing the Siddon ray-tracing method which has been widely used in clinical PET reconstruction [30]. Random, attenuation, and scatter corrections were included in the reconstruction code. The random events of every measurement were estimated by the delayed coincidence method with a delay offset of 500 ns [31]. The attenuation maps were segmented from 3D MRI images (3D Dixon in-phase/out-phase imaging sequence). Scatter correction was based on a single scatter simulation with L1-norm tail fitting [32]. All the corrections were performed in the GPU using parallel computing within several seconds. Point source measurements were performed to obtain the PSFs for resolution modeling (RM) during reconstructions. A Gaussian mixture model was used for RM to accurately capture the asymmetric PSF shapes in the reconstructed images [33]. The RM was spatially variant and was applied in the reconstruction program by a PSF operation before projection as well as a transposed PSF operation after back projection. According to the results of previous experiments, including RM in the reconstruction increased the calculation time by approximately 10% [28]. The scale of the output reconstructed image was 140 × 224 × 160, with a voxel size of 0.713 × 0.713 × 1.0 mm^3^. Design and performance specifications for the DP-PET and MRI subsystems are summarized in Table 1.

**Table 1:** Design and performance specifications for PET and MR subsystems

DP-PET		MRI	
x direction FOV	100 mm	Field strength	3 Tesla
y direction FOV	160 mm	Bore size	60 cm
z direction FOV	160 mm	B0 field homogeneity	1.16 ppm @50 cm DSV
Photodetector	SiPM	B0 field stability	< 0.1 ppm/h
Scintillator	LYSO	High order shimming	2nd order
Crystal size	15.5 × 2.76 × 2.76 mm^3^	Max gradient	45 mT/m
Number of crystal channels	3920	Max gradient slew rate	200 T/m/s
Coincidence timing window	4 ns	Number of total and independent RF channel	96/48

### Measurements

To evaluate the spatial resolution of DP-PET, a ^22^Na point source (cylinder shape with radius 0.5 mm, height 0.25 mm) with 110 kBq activity was scanned at 18 separate positions (8 min per position), including x offsets of 0, 20, and 40 mm; y offsets of 0, 20, and 40 mm; and z offsets of 0 and 40 mm in the field of view (FOV). MRI was idle during the whole scans, and the scans were repeated 3 independent times. Due to the lack of complete angle information, the filtered back projection algorithm suggested by NEMA could not be performed [34]. We instead used the proposed iterative reconstruction method (without RM) to reconstruct the point source images and referenced the assessment method in [35]. The full width at half maximum (FWHM) was determined by a Gaussian fitting. The number of iterations for the reconstruction was 100.

For sensitivity measurement, the same ^22^Na point source was scanned at y offsets of 0, 10, 20, 30, and 40 mm and z offsets of 0, 10, 20, 30, and 40 mm (30 s per position). Attenuation in the source was not taken into account for estimating the sensitivity. MRI was idle during the whole scan, and the scans were repeated 3 independent times. The random event rate was less than 5% of the true event rate, and the attenuation in the point source was neglected. The background count rate was recorded as *R*_*b*_, and the count rate of the *i*th point source measurement was recorded as *R*_*i*_. The absolute sensitivity was calculated as:

1 $${S}_i=\frac{\left({R}_i-{R}_b\right)}{\gamma A},$$

where *γ* is the branching ratio of ^22^Na, and A is the activity of the source.

For energy resolution measurement, a ^68^Ge line source with 11.4 MBq activity was scanned for 10 min. The source was positioned along the z-axis of DP-PET and was positioned at the center of the FOV. The scans were repeated 3 independent times. *Single events* were recorded to calculate energy resolution. Gaussian fitting was used for the energy spectrum of every crystal, and the result was the average energy resolution of all crystals.

We evaluate the interference between DP-PET and MRI from two different perspectives: the influence of the DP-PET insert on the MRI and the influence of the MRI on the DP-PET. To analyze the influence of the DP-PET on the MRI, an 8 × 7 × 12 cm^3^ cuboid phantom (water, NiSO_4_, NaCl) was imaged using a 3D Dixon in-phase/out-phase sequence (TR/TE = 4.91/3.19 ms, voxel size = 0.91 × 0.91 × 2 mm^3^, 549 × 384 × 164 voxels, pixel bandwidth = 1080 Hz, 39 s measurement time) with and without the DP-PET in the MR bore. The signal-to-noise ratio (SNR) and image uniformity were assessed for the MR images acquired with and without the DP-PET in the MRI bore to determine if there was any interference caused by the DP-PET on the image quality. To analyze the influence of the MRI on the DP-PET, firstly, the same ^22^Na point source was scanned at the center of the FOV (source position (0, 0, 0) mm) and the spatial resolution and sensitivity were calculated while the MRI was pulsing (3D Dixon in-phase/out-phase sequence, 8 min measurement time), and these were compared with the results obtained when the MRI was idle. The number of iterations for the reconstruction was 100. Then, a cylinder phantom (60 mm diameter and 70 mm length) filled with 757.9 kBq/mL (in total 150 Mbq) ^18^F-FDG was scanned at the center of the FOV with the axis of the cylinder phantom parallel to the z direction. The number of iterations for the reconstruction was 50. According to NEMA NU4, a 45-mm diameter (75% of active diameter) by 10-mm-long cylindrical volume of interest (VOI) was drawn over the center of the uniform region of the phantom. The PET image uniformity was calculated from the VOI while the MRI was pulsing (3D Dixon in-phase/out-phase sequence, 10 min measurement time), and these were compared with the results obtained when the MRI was idle. The experiments were repeated 3 times.

**Fig. 4:**
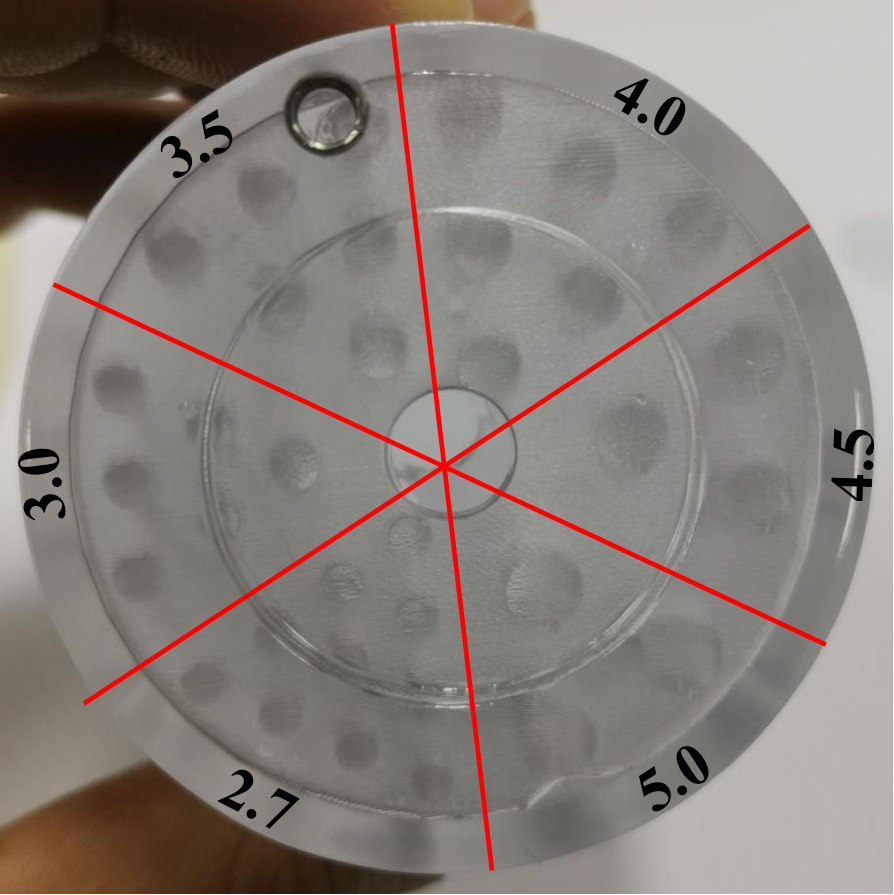
The Derenzo resolution phantom with rod diameters (in mm) indicated

To assess the spatial resolution of the DP-PET system at clinically realistic activity concentration level, a Derenzo resolution phantom [35] (3D printed with transparent photosensitive resin; shown in Fig. 4) filled with 444 kBq/mL (in total 11.1 Mbq) ^18^F-FDG was scanned. To evaluate the PET image quality, a PET scan was performed with the axis of the cylindrical phantom parallel to the z direction.

The heat-generating components of the DP-PET system mainly include FPGA boards, SiPM boards, and battery power system. To evaluate the effectiveness of the phase-change cooling system, we conducted a temperature variation experiment and a energy spectrum peak drift experiment. Specifically, a fiber optic thermometer was used to test the cooling capacity of the phase-change cooling system on the FPGA board. A DP-PET panel was assembled, and then the two fiber optic probes were placed in two key temperature monitoring areas on the FPGA board. The first was the area where the FPGA chip was located (FPGA for short), and the second was the direct current-direct current (DCDC) area where the battery power system was connected to the FPGA board. During the experiment, the power was continuously on for 45 min, and the temperature variations continued to be tracked after the power was off. As a comparison, the phase-change cooling system was taken out of the panel, and the temperature at the same position of the FPGA board without the cooling system was recorded. Measuring energy spectrum peak position drift was an indirect measurement method which reflected the temperature state of the SiPM board. As the temperature of the SiPM board rising, the energy spectrum peak would shift lower. In the test, the SiPM board worked continuously for 30 min.

## Results

### Spatial resolution

The FWHM spatial resolution values are summarized in Table 2. The spatial resolutions (average ± the standard deviation) from three independent measurements in the x, y, and z directions are shown. The spatial resolutions at the center of the FOV (source position (0, 0, 0) mm) were 2.21 mm, 2.23 mm, and 7.51 mm in x, y, and z directions, respectively. The reduced resolution in the z direction resolution is because of the limited angular coverage due to the limited size of detector panels. The spatial resolution in the z direction was uniform with z = 0 mm. However, when there was an offset in the z direction (40 mm in this example), the reduction in the spatial resolution in the z direction becomes more significant.

**Table 2:** Point source spatial resolution at different positions

Source position (x, y, z) mm	Spatial resolution (mm)		
	x	y	z
(0, 0, 0)	2.29 ± 0.01	2.36 ± 0.02	7.30 ± 0.13
(0, 20, 0)	2.20 ± 0.02	2.23 ± 0.01	7.57 ± 0.12
(0, 40, 0)	2.12 ± 0.01	2.10 ± 0.02	7.46 ± 0.07
(20, 0, 0)	2.29 ± 0.04	2.21 ± 0.06	8.07 ± 0.16
(20, 20, 0)	2.15 ± 0.04	2.23 ± 0.04	8.03 ± 0.16
(20, 40, 0)	1.97 ± 0.02	2.18 ± 0.04	8.18 ± 0.22
(40, 0, 0)	2.35 ± 0.02	2.14 ± 0.06	7.61 ± 0.47
(40, 20, 0)	2.09 ± 0.01	2.12 ± 0.02	8.49 ± 0.17
(40, 40, 0)	1.49 ± 0.03	1.58 ± 0.06	9.75 ± 0.20
(0, 0, 40)	2.24 ± 0.03	2.37 ± 0.03	9.27 ± 0.20
(0, 20, 40)	2.23 ± 0.02	2.36 ± 0.03	9.17 ± 0.16
(0, 40, 40)	2.21 ± 0.02	2.36 ± 0.02	9.47 ± 0.14
(20, 0, 40)	2.29 ± 0.03	2.34 ± 0.02	9.16 ± 0.23
(20, 20, 40)	2.27 ± 0.02	2.32 ± 0.02	9.23 ± 0.21
(20, 40, 40)	2.21 ± 0.03	2.25 ± 0.03	8.93 ± 0.15
(40, 0, 40)	1.82 ± 0.01	2.15 ± 0.01	9.37 ± 0.34
(40, 20, 40)	2.07 ± 0.02	2.13 ± 0.02	9.84 ± 0.21
(40, 40, 40)	1.97 ± 0.02	1.91 ± 0.01	9.97 ± 0.30

### Sensitivity

Table 3 shows the sensitivity % (average ± standard deviations) from three independent measurements for different positions of the point source. The sensitivity at the center of FOV (source position (0, 0, 0) mm) is 3.59%. Whether along the y-axis or z-axis, moving away from the center of FOV decreases the sensitivity of DP-PET. But, at the edge of the FOV (40 mm offset), DP-PET maintains a relatively high sensitivity of no less than 2.27%.

**Table 3:** Sensitivity at different positions

Source position (x, y, z) mm	Sensitivity
(0, 0, 0)	3.59% ± 0.03%
(0, 10, 0)	3.36% ± 0.02%
(0, 20, 0)	3.01% ± 0.01%
(0, 30, 0)	2.73% ± 0.01%
(0, 40, 0)	2.27% ± 0.01%
(0, 0, 10)	3.28% ± 0.02%
(0, 0, 20)	2.84% ± 0.02%
(0, 0, 30)	2.52% ± 0.03%
(0, 0, 40)	2.30% ± 0.01%

### Energy resolution

**Fig. 5:**
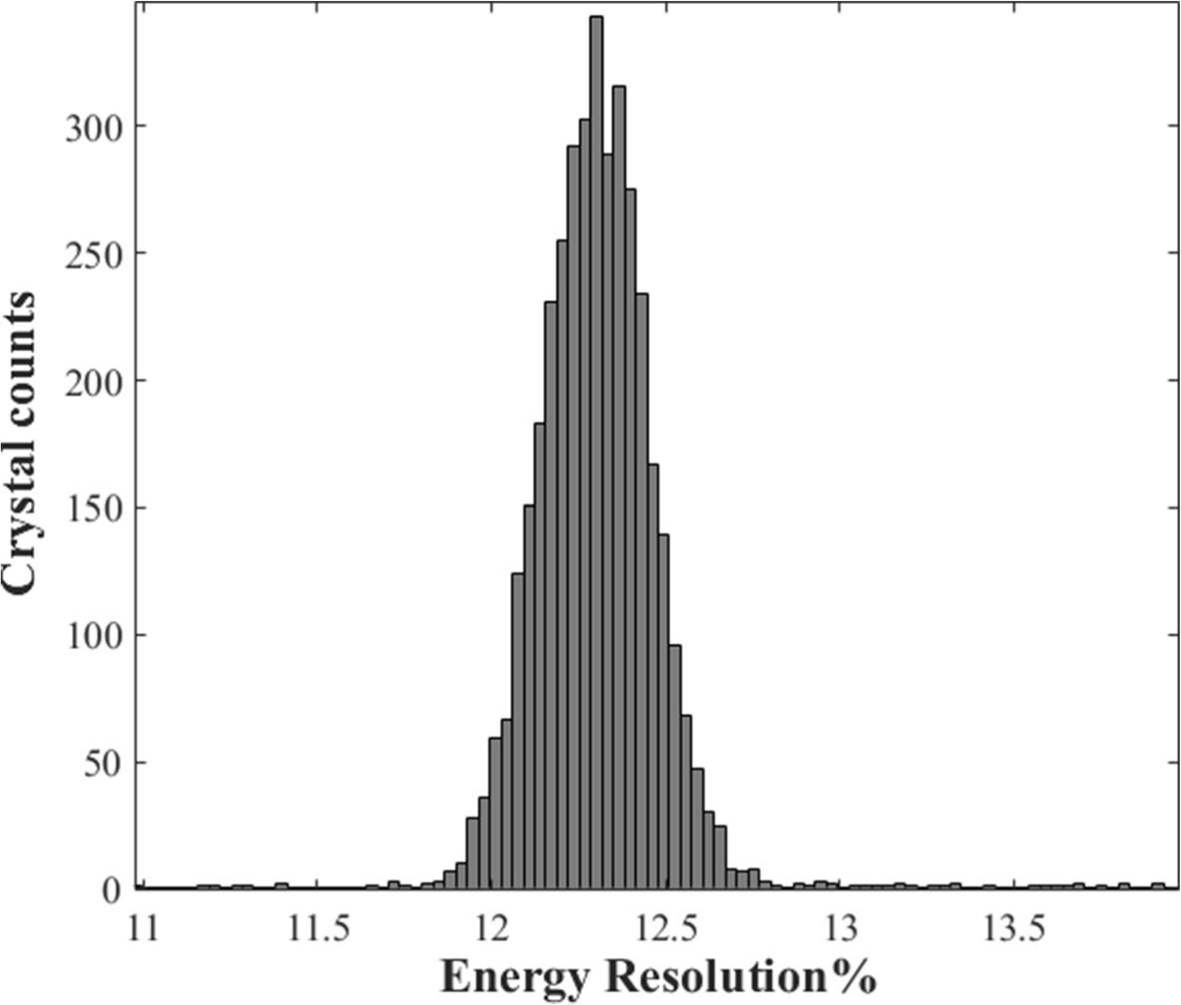
Energy resolution histogram of individual crystals

The average energy resolution % for DP-PET was calculated by taking the average of every individual crystal’s energy resolution. The results are averaged from three independent measurements. Figure 5 shows the histogram of the energy resolution for all individual crystals. The energy resolution of 94% crystals is between 12 and 13%. The average system energy resolution was measured to be 12.43% ± 0.04%.

### PET/MR interference

**Fig. 6:**
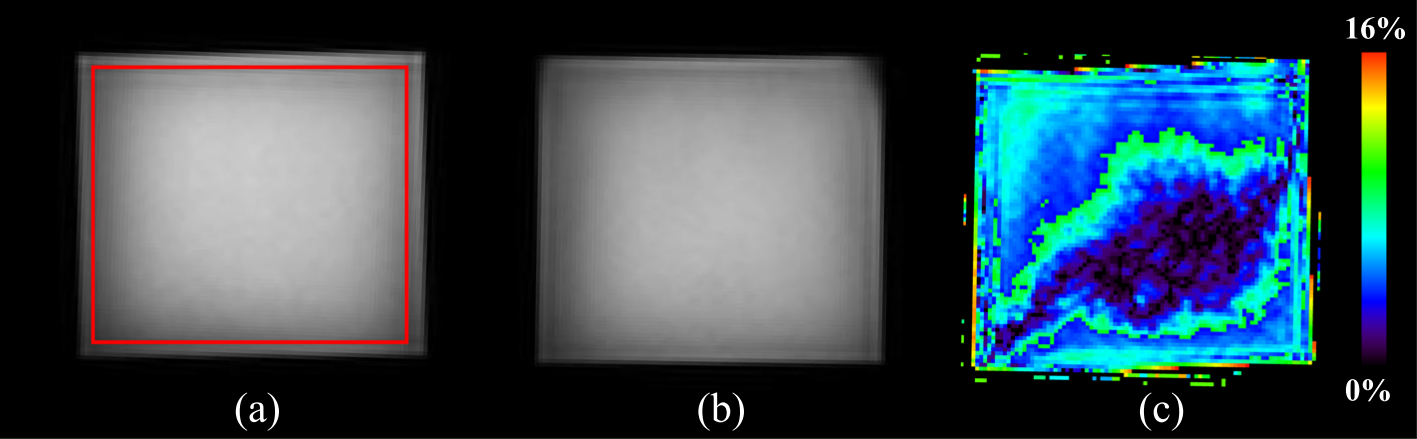
Dixon MR images with no DP-PET (**a**) and DP-PET inserted (**b**), and **c** is the result of subtracting **a** and **b**

Figure 6 shows the MRI images obtained under two different conditions (no DP-PET and DP-PET inserted) and the resulting image from subtracting them. No visible artifacts were found in the images when the DP-PET was inserted into the MRI bore. The subtraction image shows that the edges of the image suffered the most when DP-PET was inserted. In order to quantify the differences between no DP-PET and DP-PET inserted, MRI images were firstly segmented and a 3D region of interest (ROI) with at least 75% of the phantom volume was covered (Fig. 6a). The SNR within the ROI was calculated by dividing the mean value of the ROI by the standard deviation of the ROI. The MR image uniformity was accessed by using the method of normalized absolute average deviation uniformity described in the NEMA standards publication MS 3-2008 [36]. The results, shown in Table 4, were from three independent measurements with the average values and standard deviations displayed. Relative to no DP-PET inserted, when DP-PET was inserted, the mean SNR decreased by 3.88% and 1.75% for Dixon in-phase and Dixon out-phase, respectively. The image uniformity also decreased 1.01% for Dixon in-phase and 0.88% compared to when no DP-PET was present. These results suggest that putting the DP-PET in the MRI bore does not dramatically interfere with the MRI.

**Table 4:** SNR and image uniformity under different conditions

Conditions	SNR	Image uniformity
No DP-PET with Dixon in-phase	5.04 ± 0.00	83.74% ± 0.04%
DP-PET inserted with Dixon in-phase	4.85 ± 0.02	82.90% ± 0.05%
No DP-PET with Dixon out-phase	4.79 ± 0.01	83.12% ± 0.03%
DP-PET inserted with Dixon out-phase	4.70 ± 0.04	82.38% ± 0.13%

To evaluate the influence of the MR pulsing on the PET performance, Table 5 shows the spatial resolution and sensitivity while the MRI was pulsing, relative to when the MRI was idle. The spatial resolution of the point source is reduced by 1%, and the sensitivity shows a decrease below 1% under MR pulsing. There is in general less than 1% difference in spatial resolution and sensitivity with or without MR pulsing. Table 6 shows the PET image uniformity while the MR was pulsing, relative to when the MR was idle. The PET image uniformity of the VOI is reduced by 1.3% under MR pulsing. These results demonstrate that DP-PET functions properly under MR pulsing.

**Table 5:** Spatial resolution and sensitivity under different conditions

PET performance on position (0, 0 ,0) mm	MR idle	MR pulsing
Spatial resolution on the x direction (mm)	2.29 ± 0.01	2.29 ± 0.03
Spatial resolution on the y direction (mm)	2.36 ± 0.01	2.36 ± 0.03
Spatial resolution on the z direction (mm)	7.30 ± 0.13	7.32 ± 0.12
Sensitivity	3.59% ± 0.03%	3.58% ± 0.02%

**Table 6:** PET image uniformity under different conditions

	MR idle	MR pulsing
PET image uniformity of cylinder source	90.08% ± 0.64%	88.76% ± 0.58%

### PET imaging of the spatial resolution phantom

**Fig. 7:**
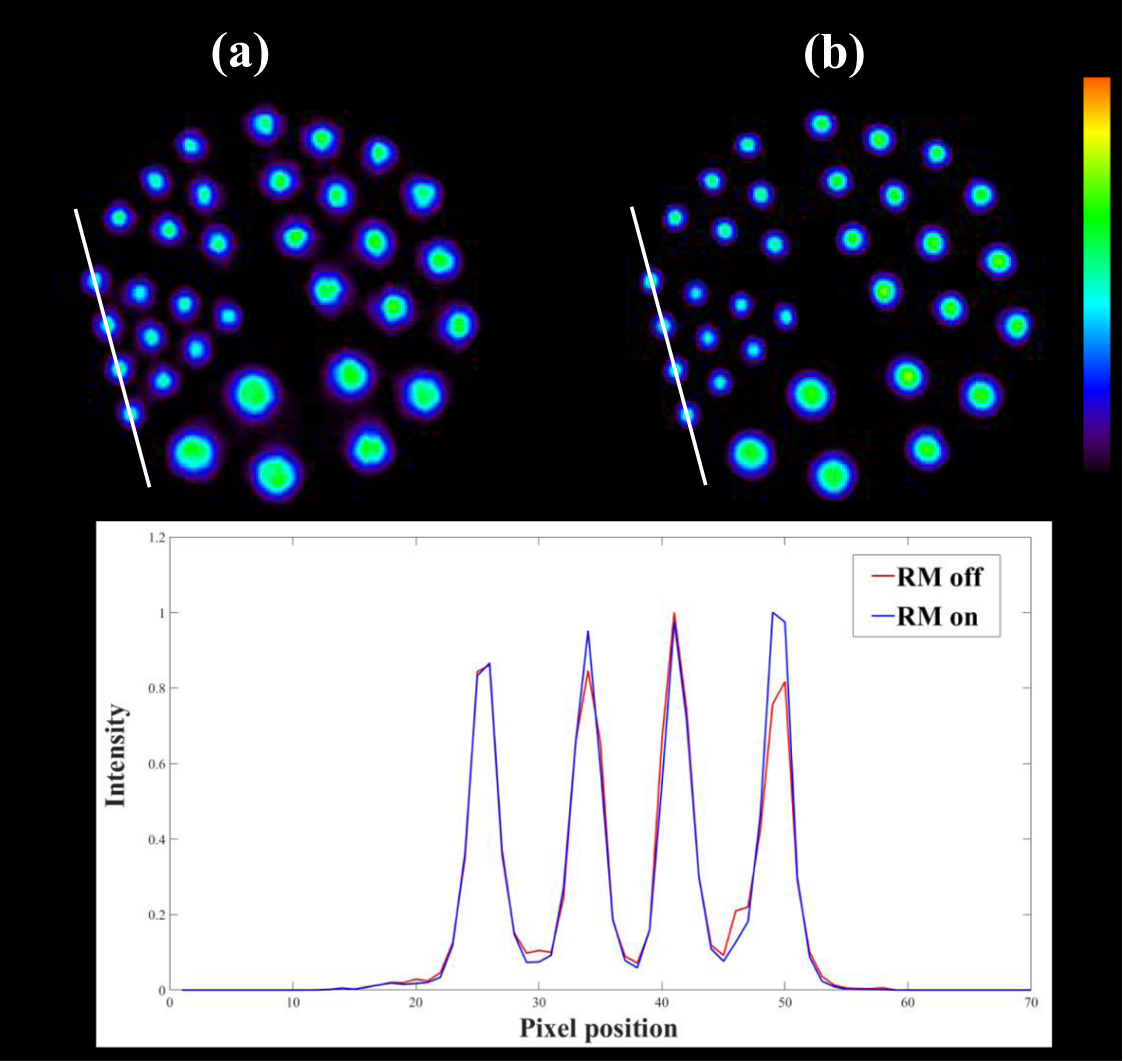
Images of the Derenzo phantom scanned parallel to the z direction with RM off (**a**) and RM on (**b**) from DP-PET scan. The bottom also shows the profiles through the hot rods, as shown by the white lines in the images

Figure 7 shows images of the Derenzo phantom scanned parallel to the z directions with RM off and on. Images were reconstructed with all corrections. The number of iterations for scatter correction was 3, and the number of iterations for reconstruction was 100. The profiles through the hot rods, as shown by the white lines in the images, are also shown in Fig. 7. The intensity of different images has been normalized to 1. All six segments are visible, and the smallest rods of 2.7 mm can be perfectly determined in Fig. 7, and the reconstruction with RM provides higher peak to valley ratios in the profiles. This demonstrates that the proposed reconstruction method with RM on can perform high spatial resolution on the x and y directions with high contrast.

### Effectiveness of phase-change cooling system

**Fig. 8:**
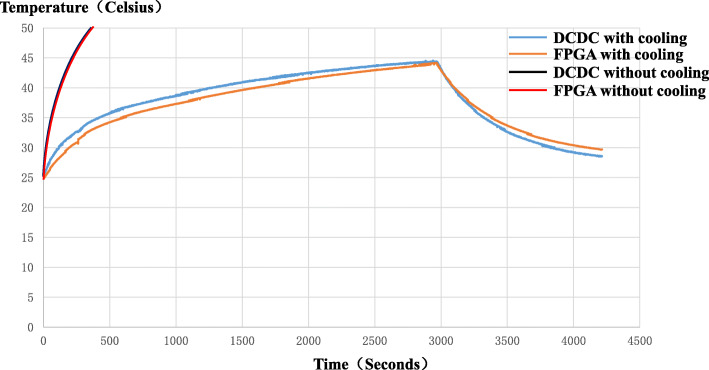
The temperature variation curve of the FPGA board with or without phase-change cooling system in different positions

Figure 8 shows the temperature variation curves of different areas on the FPGA board. When the phase-change cooling system was removed, the temperature of the FPGA area and DCDC area rose quickly, and the temperature of the optical fiber thermometer reached 50 °C within 5 min. After adding the phase-change cooling system, the temperature in the FPGA area and the DCDC area rose significantly slower. This demonstrated that the phase-change cooling system of the DP-PET system can effectively absorb the heat generated by the FPGA board.

**Fig. 9:**
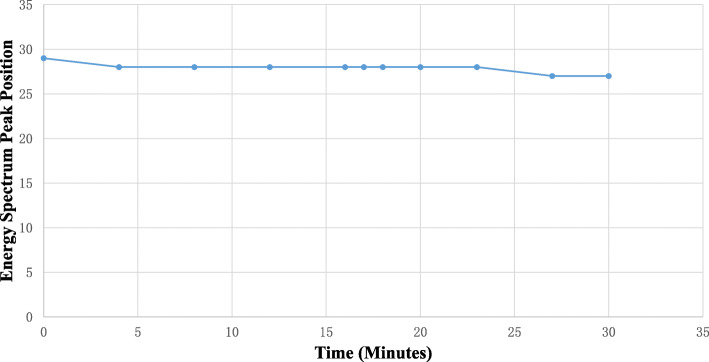
The energy spectrum peak position of the DP-PET system over time

Figure 9 shows the result of the energy spectrum peak shift of the DP-PET system. As time increases, the energy peak continuously shifted to 3% lower than its starting value after 30 min of operation. The temperature measurement and energy spectrum peak drift evaluation demonstrate that the phase-change cooling material can efficiently absorb a large amount of heat generated by the FPGA and SiPM boards.

## Discussion

In this work, we developed a portable MR-compatible PET insert and evaluated its performance inside a clinical 3-T MRI scanner. Measurement results of the DP-PET demonstrated high resolution and sensitivity during simultaneous PET/MR acquisition. Because of its unique battery power pack and cableless cooling design, the DP-PET can potentially be used for a large variety of clinical and research applications. For instance, without additional hardware modification, a clinical MRI scanner can achieve simultaneous PET/MR imaging for targeted organs such as the brain and breast.

We chose dual-panel breast imaging as the first configuration for verification of DP-PET. In future pre-clinical research, patients will lie on the MRI bed with the breast vertically downward and the DP-PET system will be fixed on both sides of the breast. Mammography and ultrasound are the most common methods for diagnosis and guided intervention in breast disease. MRI has also become an increasingly important method in breast cancer diagnosis and post-operation examination [37]. Breast-dedicated PET imaging systems have been developed in recent years and demonstrate clinical feasibility [38–40]. The dual-panel geometry in breast-dedicated PET has been widely used due to the advantage of lower costs and potentially increased sensitivity [41–43]. There is a growing interest in developing breast PET/MR, and some simulation studies as well as prototypes have proved the increased sensitivity and specificity in the detection of small lesions [44, 45]. In considering biopsy applications in the breast, a dual-panel detector configuration is more practical than a ring detector for the convenience of breast immobilization [46]. Our study demonstrated that the proposed DP-PET insert prototype maintained high performance inside a strong magnetic field which shows great promise for breast PET/MR applications. In our future research, a different geometric configuration such as cylindrical configuration for brain imaging will be explored.

There are a few limitations to this study. Because there is not a matching MRI breast coil, all images were collected using whole-body transmit/receive coil which might achieve sub-optimal MRI image quality. We are still working on developing a dedicated breast coil for DP-PET. An ideally designed breast coil should not affect the uniformity of the main magnetic field, and the coil should be designed to be gamma transparent [47]. When considering the flexibility of the DP-PET, the coil should allow for variable spacing and be exchangeable. In this work, we used the Derenzo phantom with no background activity. We acknowledge that this was a limitation and more accurate spatial resolution, and contrast measurements could be obtained if the phantom had a background activity. Moreover, the proposed DP-PET was a prototype system, and therefore, there was limited testing of the battery power supply robustness.

## Conclusion

In conclusion, we developed a MR-compatible PET insert prototype and performed several studies to begin to characterize the performance of the proposed DP-PET and presented the initial results here. Measurements of PET performance demonstrate that DP-PET has a spatial resolution better than 2.5 mm (parallel to the detector panels) and a maximum sensitivity of 3.59% in the center of FOV. In addition, the energy resolution was measured to be an average of 12.43%. We also studied the interference between DP-PET and 3-T MRI system. The results showed that the proposed DP-PET could work optimally in the MRI bore and would cause little influence on the MRI images. Finally, a Derenzo phantom was scanned in DP-PET. With all corrections and RM applied, high-quality images were obtained using the proposed GPU-based reconstruction method.

## Data Availability

The datasets used and analyzed during the current study are available from the corresponding author on reasonable request.
